# Hyperglycemia abolishes the protective effect of ischemic preconditioning in glomerular endothelial cells in vitro

**DOI:** 10.14814/phy2.12346

**Published:** 2015-03-24

**Authors:** Katie J Schenning, Sharon Anderson, Nabil J Alkayed, Michael P Hutchens

**Affiliations:** 1 Department of Anesthesiology and Perioperative Medicine, Oregon Health and Science UniversityPortland, Oregon; 2 Division of Nephrology and Hypertension, Department of Internal Medicine, Oregon Health and Science UniversityPortland, Oregon; 3 Department of Internal Medicine, Portland Veterans Affairs Medical CenterPortland, Oregon; 4 Knight Cardiovascular Institute, Oregon Health and Science UniversityPortland, Oregon

**Keywords:** Glomerular endothelial cells, hyperglycemia, ischemic preconditioning, renal ischemia–reperfusion injury

## Abstract

In preclinical investigations, ischemic preconditioning (IPC) protects kidneys from ischemia/reperfusion injury. The direct effects of IPC on glomerular endothelial cells have not been studied in detail. Most investigations of IPC have focused on healthy cells and animals, and it remains unknown whether IPC is renoprotective in the setting of medical comorbidities such as diabetes. In this study, we determined the preventive potential of IPC in healthy glomerular endothelial cell monolayers, and compared these results to monolayers cultured under hyperglycemic conditions. We exposed glomerular endothelial monolayers to 1 h of IPC 24 h prior to oxygen–glucose deprivation (OGD), an in vitro model of ischemia/reperfusion injury. Glomerular endothelial monolayer integrity was assessed by measuring transendothelial electrical resistance, albumin flux, and cell survival. We found that IPC protected healthy but not hyperglycemic glomerular endothelial monolayers from ischemia/reperfusion injury. Furthermore, not only was the protective effect of IPC lost in the setting of hyperglycemia, but IPC was actually deleterious to the integrity of hyperglycemic glomerular endothelial cell monolayers.

## Introduction

Acute kidney injury (AKI) is a common complication in hospitalized patients, and is associated with a mortality rate as high as 70% in critically ill patients (Chertow et al. 1998; Lassnigg et al. 2004; Uchino et al. 2005). In patients with AKI who require renal replacement therapy, 10% progress to dialysis-dependent end-stage renal disease (Triverio et al. 2009). The most common cause of AKI is renal ischemia/reperfusion injury (IRI), and both experimental and clinical data have shown that hyperglycemia and diabetes increase the risk of renal IRI (Goor et al. 1996; Melin et al. 1997; Shi et al. 2007; Parekh et al. 2011).

Although renal proximal tubular injury is considered the hallmark of AKI pathology, recent evidence suggests that complex molecular and cellular interactions among tubular epithelium, the glomerular filtration barrier, and inflammatory mediators contribute to AKI (Akcay et al. 2011; Lee et al. 2012; Xu et al. 2014). The glomerular endothelium is a critical part of the glomerular filtration barrier, and the integrity of this barrier prevents the leak of albumin and other proteins into the urine. Renal ischemic injury leads to dysfunction of the glomerular filtration barrier marked by microvascular permeability and proteinuria (Andersson et al. 2007; Wagner et al. 2008; Hutchens et al. 2012). Similarly, glomerular endothelial dysfunction in the setting of hyperglycemia and diabetes is becoming increasingly recognized as a major factor in the development of microalbuminuria and diabetic nephropathy (Satchell and Braet 2009; Salmon and Satchell 2012; Weil et al. 2012).

There are no effective therapeutic strategies for AKI. Experimentally, ischemic preconditioning (IPC) has shown success in conferring protection against renal IRI in animal models (Lee and Emala 2000; Ogawa et al. 2000; Park et al. 2001), but this success remains to be seen in clinical trials. In vitro, IPC has produced ischemic tolerance in cardiomyocytes, neurons, and proximal tubular epithelial cells (Turman and Bates 1997), but little is known regarding the effects of IPC in glomerular endothelial cells.

Expanding on our previously developed model of IRI in cultured glomerular endothelial cells (Hutchens et al. 2012), we developed an in vitro model of IPC to test our hypothesis that IPC would protect glomerular endothelial cells (GenC) from a model of IRI. Furthermore, although there is evidence from the cardiac literature showing that hyperglycemia abolishes the protective effects of IPC (Kersten et al. 1998, 2000), little is known regarding the role of hyperglycemia in renal IPC. Therefore, the purpose of this study was to evaluate our hypothesis that hyperglycemia would negate the effects of IPC in glomerular endothelial cells.

## Materials and Methods

### Glomerular endothelial cell (GenC) culture

This conditionally immortalized murine glomerular endothelial cell line was developed in the H-2Kb-tsA58 immortomouse by Dr. Michael Madaio (Akis and Madaio 2004), Temple University and was a gift of Dr. H. Abboud, University of Texas, San Antonio. GenC were cultured in the proprietary microvascular endothelial growth medium-2 (EGM2-MV Bullet Kit, Lonza; Walkersville, MD) containing 5% fetal bovine serum (FBS), human fibroblast growth factor-2, vascular endothelial growth factor, R^3^-insulin-like growth factor-1, human endothelial growth factor, hydrocortisone, ascorbic acid, and gentamicin–amphotericin. GenC were grown to confluence on membrane support inserts with 0.4 *μ*m pores (Transwell, Corning, Corning NY). Medium was changed daily, and transendothelial electrical resistance (TEER) was measured daily. When the TEER was greater than 30 Ω × cm^2^, cells were considered ready for experimentation. Each experiment was performed in triplicate at a minimum.

### Glycemic conditions

The standard medium, described above, contains 5.5 mmol/L D-glucose. High glucose conditions were achieved by adding D-glucose to the standard medium described above in order to achieve a concentration of 25.5 mmol/L D-glucose. For normal glucose conditions, mannitol was incorporated into the standard medium as an osmotic control (5.5 mmol/L glucose + 20 mmol/L mannitol). Media for all experiments were maintained at 300 ± 20 mOsm.

### Ischemia/reperfusion injury model

We have previously established an in vitro model of IRI (Merkel et al. 2010) which involves subjecting cultured GenC to ischemic injury induced by oxygen–glucose deprivation (OGD) followed by a period of reoxygenation/glucose repletion (RGR). Briefly, GenC monolayers were subjected to 8 h of oxygen–glucose deprivation (OGD) after which medium was replaced using the standard growth medium and the cells were returned to the incubator for a 12-h period of RGR.

### Ischemic preconditioning model

To create an in vitro model of ischemic preconditioning, GenC monolayers underwent 1 h of OGD followed by 24 h of RGR. In this model of late ischemic preconditioning, the IPC stimulus was performed 24 h prior to exposing cells to the model of IRI described above. While the effects of early preconditioning are typically present immediately after the ischemic stimulus and dissipates after 2–3 h, late preconditioning generally occurs 12–24 h after the stimulus and lasts for up to 72 h (Tomai et al. 1999).

### Transendothelial electrical resistance

Transendothelial electrical resistance (TEER) across the monolayer was measured using an endothelial microvolt-ohmmeter and concentric-ring electrode system (EndOhm, World Precision Instruments; Sarasota, FL). Measurements were performed daily to assess readiness for experimentation, as well as following periods of IPC, OGD, and RGR. After the cell support insert was placed in the ohmmeter, readings were allowed to stabilize for 10 sec before recording a final value. The resistance of a cell-free insert was subtracted from each measurement, and the resulting value was multiplied by the surface area of the membrane support to obtain TEER in Ω × cm^2^. The final TEER value prior to experimentation was considered the “baseline” value, and results are reported as a percentage of baseline.

### Transendothelial protein passage

Transendothelial permeability to albumin was determined by measuring passage of fluorescein isothiocyanate (FITC)-labeled albumin (Sigma-Aldrich, St. Louis, MO) across the cell monolayer. Following OGD, FITC-albumin was added to the cell insert. Following 4 and 8 h of RGR, 100 *μ*L of media from each lower chamber of the membrane support system was aspirated and placed into one well of a 96-well plate. FITC-Albumin fluorescence was then measured using a plate reader (VICTOR3, PerkinElmer, Waltham, MA) with excitation and emission wavelengths of 405 and 535 nmol/L, respectively. The amount of FITC-albumin passing through the monolayer is expressed in arbitrary units of FITC fluorescence.

### Assessment of cell viability

The morphology of cells subjected to OGD/RGR was examined using phase contrast microscopy. To determine cell viability the colorimetric 3-(4,5-dimethylthiazol)-2,5-diphenyl tetrazolium bromide (MTT) metabolic activity assay was used. Following treatments, 1 mg/mL MTT (Sigma) was added to each cell culture well and the plate was incubated at 37°C for 4 h. After removing supernatant from each well, formazen crystals were lysed in DMSO by gently shaking the plate. Absorbance was measured at 570 nmol/L using a microplate reader (VICTOR3, PerkinElmer). Cell viability was expressed as a percentage of the value in the control (untreated) cultures.

### Statistical analysis

Analysis was performed using Prism 5 software (GraphPad; San Diego, CA). Two group comparisons of TEER values were performed using paired *t*-tests with two-tailed p values, and comparisons of transendothelial protein flux were performed using unpaired *t*-tests with two-tailed *P* values. Cell viability comparisons were performed using one-way analysis of variance (ANOVA). Analysis of the change in TEER values over time was performed using a one-way ANOVA for repeated measures. Data are presented as mean ± SEM. Probability statistic *P *< 0.05 was taken to indicate significance.

## Results

### OGD causes GenC monolayer dysfunction

**Figure 1:**
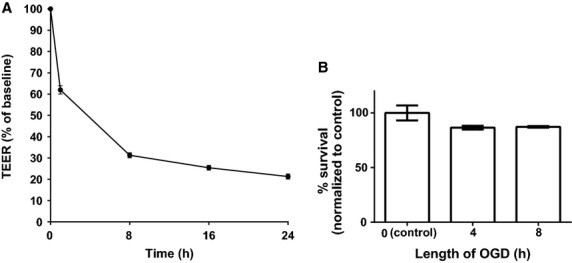
(A) Graph showing transendothelial electrical resistance (TEER) recordings of GenC monolayers under normal glucose conditions. TEER (*y*-axis) is shown as a percentage of baseline recordings over time (*x*-axis). Results show that Oxygen–Glucose Deprivation (OGD) causes GenC monolayer dysfunction that was maximal in the first 2 h of OGD and remained rapid for the first 8 h (*n *= 5, *P* < 0.0001). (B) Graph showing percentage of surviving GenC normalized to normoxic controls (*y*-axis) following exposure to OGD over time (*x*-axis). Results show that OGD does not cause an increase in GenC cell death compared to normoxic controls (*n* = 3, *P* = 0.13).

To determine the effect of OGD on GenC monolayer function, we first measured the TEER of GenC monolayers after exposure to OGD for periods ranging from 1–24 h. OGD exposure significantly decreased GenC monolayer integrity as evidenced by decreases in TEER. The loss of monolayer integrity declined rapidly in the first 8 h, and was followed by a much more gradual decrease for the next 16 h (Fig.1A, *n* = 5, *P* < 0.0001). We then used the MTT assay to determine whether this decrease in TEER corresponded with cell death. The proportion of cell death in GenC monolayers exposed to OGD was not significantly different than the cell death observed in control GenC monolayers maintained in normoxic conditions (Fig.1B, *n* = 3, *P* = 0.13).

### Hyperglycemia causes GenC monolayer dysfunction under normoxic conditions

**Figure 2:**
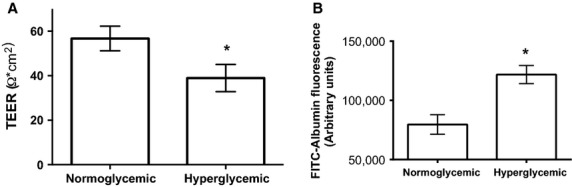
(A) Graph of TEER of GenC monolayers cultured in either normal glucose or high glucose conditions. Results show that high glucose causes a decrease in TEER (*n* = 5, *P* < 0.0001). (B) Graph of albumin flux across GenC monolayers cultured in normal or high glucose conditions. Results show that high glucose causes an increase in albumin flux (*n* = 3, *P* = 0.02).

To determine the effect of prolonged hyperglycemia on GenC function in normoxic conditions, GenC were cultured for 7 days in hyperglycemic or normoglycemic, osmotically controlled conditions. Normoglycemic GenC had significantly higher TEER (mean ± SEM, 57 ± 5.6) when compared with hyperglycemic GenC (39 ± 1 Ω, *n* = 5, *P* < 0.0001, Fig.2A). Furthermore, hyperglycemia caused increased GenC monolayer permeability to albumin, as illustrated in Fig.2B (*n* = 3, *P* = 0.02).

### Hyperglycemia increases GenC susceptibility to IRI

**Figure 3:**
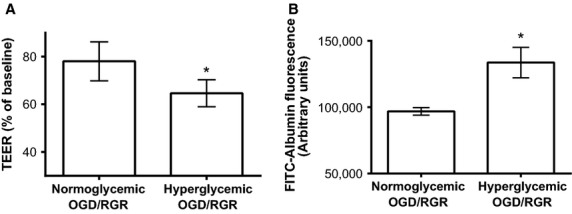
Following 8 h of OGD and 12 h of RGR, there was (A). decreased TEER in hyperglycemic compared to normoglycemic GenC monolayers (*n* = 5, *P* = 0.02), and (B). increased albumin flux across hyperglycemic compared to normoglycemic GenC monolayers (*n* = 3, *P* = 0.04).

We found that not only was hyperglycemia detrimental to GenC monolayer integrity under normoxic conditions, as described above, but hyperglycemia also increased the susceptibility of GenC monolayers to a model of IRI. To test the resilience of normoglycemic versus hyperglycemic monolayers following a model of IRI, both groups of GenC were exposed to 8 h OGD followed by 24 h RGR. The function of hyperglycemic GenC following OGD/RGR was 64.6% of baseline compared to control, normoglycemic GenC, which maintained 78.1% of their baseline function following OGD/RGR (Fig.3A, *P* = 0.02, *n* = 5). Similarly, hyperglycemic GenC monolayers had increased permeability to albumin following OGD/RGR when compared with normoglycemic GenC (Fig.3B, *n* = 3, *P* = 0.04).

### IPC protects normoglycemic GenC but exacerbates hyperglycemic injury to GenC

**Figure 4:**
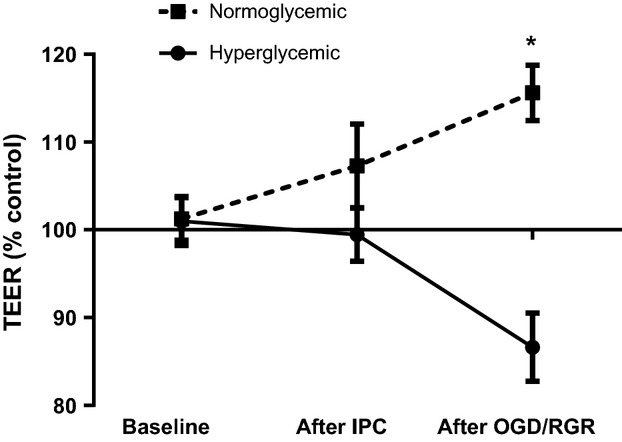
Graph showing TEER of normoglycemic and hyperglycemic GenC exposed to ischemic preconditioning (IPC) prior to OGD/RGR. TEER values are represented as percentage of control GenC that were exposed to OGD/RGR but not exposed to IPC. Results show that IPC protects normoglycemic GenC function (*n* = 4, *P* = 0.01) but makes hyperglycemic GenC more susceptible to OGD/RGR-induced injury (*n* = 4, *P* = 0.03).

To determine the effect of a model of IPC on GenC function, 24 h prior to exposure to OGD/RGR GenC were exposed to either 1-h of IPC or sham IPC (media changes but no OGD). As illustrated in Fig.4, when compared with controls (normoglycemic GenC + sham IPC) normoglycemic GenC that underwent an in vitro model of IPC had increased TEER following OGD/RGR (*n* = 4, *P* = 0.01). Prolonged hyperglycemia not only negated the protective effect of ischemic preconditioning, but it actually had a deleterious effect (*n* = 4, *P* = 0.03) on hyperglycemic GenC when compared with controls (hyperglycemic GenC + sham IPC).

## Discussion

The main novel findings demonstrated in this study are (1) an in vitro model IPC protects the integrity of normoglycemic GenC monolayers when exposed to a model of IRI, and (2) the protective effect of IPC was not only abolished in the setting of prolonged hyperglycemia, but IPC was actually detrimental to GenC monolayer function compared to cells that were not exposed to IPC.

Our finding that hyperglycemia increases GenC permeability supports an in vivo study by Axelsson et al. (2010) showing that hyperglycemia increased glomerular permeability in rats and an in vitro investigation by Singh and coinvestigators in which hyperglycemia increased the passage of albumin across GenC monolayers (Singh et al. 2011). However, although we found a decrease in TEER across GenC monolayers secondary to hyperglycemia, Singh et al. (2011) did not show a difference in TEER values. This difference might be explained by their use of a human cell model and our use of a mouse cell model.

IPC is an effective renoprotective strategy in mice and rats in vivo, and in tubular epithelial cells in vitro (Turman and Bates 1997; Lee and Emala 2000; Ogawa et al. 2000; Park et al. 2001). Our results suggest that the same is true in glomerular endothelial cells in vitro. Despite these encouraging laboratory investigations, IPC for renal protection has not yet been translated successfully into clinical practice. The reason that IPC has not yet made it to the bedside is likely multifactorial. One potential barrier to translation is that the majority of ischemic preconditioning studies have been performed in healthy cell or animal models. Clinically, patients with diabetes are more prone to ischemia–reperfusion injury (Sanada et al. 2011; Parolari et al. 2012). In this study, we showed that in vitro, the protective effect of ischemic preconditioning on glomerular endothelial cells is abrogated in the setting of prolonged hyperglycemia. These data are in accord with those reported by Kersten and colleagues who have shown that, in the heart, the protective effect of ischemic preconditioning is abolished in diabetic animals (Kersten et al. 1998, 2000; Balakumar et al. 2009). Before IPC for renal protection enjoys widespread clinical use, further investigation is needed regarding the effects of IPC on patients with comorbidities such as diabetes.

Our study has limitations, and we recognize the limitations inherent of an in vitro model. Although an in vitro model of prolonged hyperglycemia is not equivalent to an in vivo model of diabetes, it should be noted that not only are diabetic animals at increased risk of IRI, but nondiabetic animals with elevated blood glucose levels have also been found to be more vulnerable to IRI (Moursi et al. 1987; Podrazik et al. 1989). Our experiments were performed in an immortalized murine GenC line, and these cells are likely more resistant to ischemic injury than primary cells. Thus, it may be beneficial to repeat these experiments in primary human GenC. Additionally, GenC are only one component of the greater glomerular filtration barrier, and a coculture model that includes podocytes might help to inform future in *vivo* studies (Byron et al. 2014).

Elucidation of the molecular details underlying the protective effects of IPC will help to develop preventive and therapeutic strategies and will help clarify how hyperglycemia negates this protection. Vladic et al. (2011) determined that the dysregulation of endothelial nitric oxide synthase by hyperglycemia impairs the cardioprotective effect of IPC. Studies have implicated nitric oxide (Park et al. 2003; Yamashita et al. 2003), mitogen-activated protein kinases (Park et al. 2001), protein kinase C, and G proteins (Lee and Emala 2001) in the mechanism of IPC-induced renal protection.

AKI is associated with increased morbidity, mortality, and cost of care, and there are currently no effective prevention strategies. IPC affords renoprotection from IRI in vivo, and we have now shown that IPC renders GenC refractory to an in vitro model of IRI. Additionally, we are the first to demonstrate that the resistance afforded GenC by IPC is abolished in the setting of prolonged hyperglycemia. Further elucidation of the underlying mechanisms contributing to this phenomenon will assist in developing clinically relevant protection modalities.

## Conflict of Interest

None declared.
